# Ultrasound Versus Fluoroscopy for Catheter Tip Confirmation in Long-Term Vascular Access: A Prospective Observational Study

**DOI:** 10.7759/cureus.86604

**Published:** 2025-06-23

**Authors:** Mary Tina Rani G Vaz, Nitish Vijayanand, Jujju Jacob Kurian, Leni Grace Mathew, Ekta Rai, Jubin Merin Jacob, Grace Rebekah

**Affiliations:** 1 Department of Anaesthesiology, Christian Medical College, Vellore, Vellore, IND; 2 Department of Paediatric Surgery, Christian Medical College, Vellore, Vellore, IND; 3 Department of Child Health, Christian Medical College, Vellore, Vellore, IND; 4 Department of Biostatistics, Christian Medical College, Vellore, Vellore, IND

**Keywords:** central vascular catheter, echocardiography, fluoroscopy, pocus, radiation

## Abstract

Study design and objective: A prospective observational study aimed to study the use of ultrasonographic location of the catheter tip as an alternative to on-table fluoroscopy in determining the correct placement of long-term vascular access devices (VADs) in children.

Methods: The study was conducted in a large, tertiary care, teaching hospital in South India. A total of 30 consecutive paediatric haemato-oncology patients were planned for surgical insertion of long-term VADs (Hickman^TM^ catheter (Bard Access Systems, Salt Lake City, UT, USA) or Port-a-Cath^®^ (Smiths Medical, St. Paul, MN, USA)), recruited over a period of three months. The primary outcome of the study was to see if the ultrasonographic location of the catheter tip was in agreement with on-table fluoroscopy findings.

Procedure: After surgical insertion of the long-term VAD, an intra-operative trans-thoracic ultrasonography of the heart was done prior to fluoroscopy. A four-chamber view of the heart was obtained using either the sub-xiphoid or apical view. With the heart chambers in view, 2 mL of saline was injected rapidly into the external port, and the corresponding turbulence was visualized in real-time on transthoracic echocardiography (TTE). Depending on the site of appearance of the turbulence, the catheter tip location was inferred (right atrium (RA), above the RA, or in the right ventricle (RV)). The location of the catheter tip was then verified using fluoroscopy. ​​​​Kappa statistics were calculated to obtain the degree of agreement between ultrasound and fluoroscopy.

Results: We found a 92.5% overall agreement between ultrasound and fluoroscopy, which was significant (p-value <0.01). The degree of agreement was 100% for the RV positions, while it was 95.5% for the RA position, and 83.3% for the above RA.

Conclusion: Ultrasonographic confirmation of the vascular catheter tip position is a quick, easy-to-perform, cost-effective, and safe alternative to fluoroscopy.

## Introduction

Long-term vascular access devices (VADs) in children are indicated in those requiring chemotherapy, antibiotics, total parental nutrition, or fluid replacement therapy for more than three months. In our institution, these devices are inserted surgically, under anaesthesia, with the position of the catheter tip confirmed on the table by fluoroscopy. Though fluoroscopy is considered the gold standard reference modality for confirming the position of the catheter tip, it has the downside of exposing the child to harmful ionizing radiation. Radiation exposure may have a significant impact on children when compared to adults due to their higher sensitivity to ionizing radiation [1-3], and a higher tendency to accumulate radiation over a longer expected lifespan, resulting in increased susceptibility to malignancies in later life [4].

Also, fluoroscopy mandates the use of a portable image intensifier with a qualified technician and a compatible operating table. Organizing all these for confirming the position of the VAD catheter tip (usually a short-duration procedure) has often resulted in a long wait time, which in turn may have had a significant short-term impact on a high-volume centre in the form of cancellation of the last one or few cases. As an alternative, we thought of using sonography as a diagnostic modality for confirming the position of the VAD catheter tip. However, literature on the same has been sparse. Prekker et al. in 2010 were among the first to attempt the use of ultrasound in confirming the position of the catheter tip [5]. Following this, there have been a few other studies conducted on adult [6,7] and paediatric patients [8], with polyurethane central venous catheters that are intermediate-term non-tunneled vascular catheters [9]. These studies found the indirect "saline flush test" with ultrasonography to be a reliable alternative to fluoroscopy [10].

However, similar studies have not been done on long-term, tunneled, polyurethane catheters, where the location of the catheter tip is extremely important [11]. In our study, we attempt to evaluate the efficacy of ultrasound in confirming the location of the catheter tip of long-term VAD in haemato-oncology patients.

## Materials and methods

This prospective observational study was conducted in a large, tertiary care, teaching hospital (Christian Medical College and Hospital, Vellore, Vellore), in South India after obtaining approval from the Institutional Review Board (No. 10861 dated 27.09.2017). After calculating the sample size, 30 consecutive paediatric hematology and oncology patients posted for long-term VAD (cuffed tunneled Hickman^TM^ catheter (Bard Access Systems, Salt Lake City, UT, USA) and Port-a-Cath​​​​^®^ (Smiths Medical, St. Paul, MN, USA), respectively) placement were enrolled in the study between January 2018 and April 2018. Informed consent was obtained from the parents by one of the investigators the night before the procedure, and informed assent was taken from children 12 years and above as per the institutional protocol. Fasting and premedication orders were administered as per standard practice.

All participants were administered general anaesthesia with endotracheal tube placement. The patient was positioned supine with a roll under the shoulder and the neck turned to the side opposite to the side of catheter insertion to provide good access. A scout ultrasound scan using a General Electric (GE) cardiac (1-5 MHz, B mode) was then done to ensure that a good four-chambered cardiac view was obtained either through the sub-costal or apical window. Following this, surgical insertion of the VAD catheter into the internal jugular vein was performed. After ensuring free back flow of blood, the scrubbed principal investigator obtained a four-chamber view in a strict aseptic manner. Ventilation was briefly interrupted (<5 seconds) in order to improve the quality of the scanned images. Once a good view was obtained, the surgeon rapidly injected 2 mL of saline into the external injection port using a 2-cc syringe. The shape of opacification caused by the turbulence of injection and the time to appearance of the jet were noted with a stopwatch. Saline injection was repeated till a good image was captured, with some requiring two to three injections due to the perceived lack of coordination between the surgeons and the image saver. The findings were then documented, and an inference was made as to where the catheter tip was situated (Table 1). Absence of flow, a trickle from above, or a delayed appearance of the jet (more than two seconds) denoted a high malposition. A triangular jet or complete opacification in the right atrium (RA) meant that the catheter was in the correct position. Catheters sited deep in the RA had the apex of the triangular jet shifted down. Opacification of the right ventricle (RV) first meant that there was a low malposition. After documenting the position by ultrasound, the chief surgeon, who was blinded to the result, would proceed with fluoroscopy to determine the position. The findings were then corroborated, and the decision to withdraw or insert the catheter further was based on the fluoroscopic image. The tip of the catheter lying at the level of carina (cavo-atrial junction) [12] or just below it (RA) was deemed acceptable [13], whereas a tip above the carina was considered unacceptable, as it would indicate the tip was in the superior vena cava, thus making it prone to thrombosis [11].

**Table 1:** Characteristics of turbulence RA: right atrium; RV: right ventricle; SVC: superior vena cava; PA: pulmonary artery

Characteristics of turbulence		Inference
Pattern	No turbulence	Arterial location of the catheter
	Starry appearance	Catheter tip located high in the SVC
	Opacification with proximal apex	SVC-RA junction
	Opacification	Located in RA/RV, depending on the chamber opacified
Site	RA	Proximity to RA
	RV	RV
	No opacification	Either high in SVC or very deep in PA
Timings	<2 secs	In the same chamber (RA/RV) or in close vicinity
	>2 secs	High above the chamber

Continuous data was presented as mean ± standard deviation, and categorical data was presented as frequencies and percentages. Statistical analysis was performed using IBM SPSS Statistics for Windows, Version 21 (Released 2013; IBM Corp., Armonk, New York, United States). Weighted inter-rater agreement between the two modalities was also calculated.

## Results

We recruited 30 children for the study. Their demographic details are summarized in Table 2. All patients underwent placement of a VAD under general anaesthesia.

**Table 2:** Demographic characteristics IJV: internal jugular vein; TTE: transthoracic echocardiography; VAD: vascular access device

Characterstic	Mean (SD)	Number (%)
Age (years)	5.2 (4.4)	-
Gender		
Male	-	19 (63.3)
Female	-	11 (36.7)
Height (cm)	106 (28.9)	-
Weight (kg)	19 (13.6)	-
Diagnosis		
Solid tumors	-	3 (10)
Benign hematological pathology	-	12 (40)
Malignant hematological pathology	-	15 (50)
VAD		
Port-a-Cath	-	17 (56.7)
Cuffed tunneled Hickman catheter	-	13 (43.3)
VAD insertion site		
Right IJV	-	29 (96.7)
Left IJV	-	1 (3.3)
TTE		
Subxiphoid view	-	28 (93.3)
Apical view	-	2 (6.7)

Placement of the catheter tip at the cavo-atrial junction or the proximal RA was considered optimal, with the catheter to be positioned parallel to the blood flow rather than perpendicular. The time taken for the saline flush to appear was less than two seconds for 27 VADs and two seconds or more in three patients.

Ultrasonography revealed eight catheter tips to be suboptimally positioned, five of which were high and three were low (Table 3). Of the five high ones, the assessor documented the need to push in three catheters based on abnormal turbulence shape and timing, whereas two catheters with an abnormal turbulence had a normal timing of within two seconds. Fluoroscopy done on the five high catheters (diagnosed ultrasonographically) revealed four catheters to be high, requiring them to be pushed in, and one at the cavo-atrial junction, for which no manipulation was done. Three catheters were found to be in the RV, both by ultrasound (opacification of the RV first with a timing less than two seconds) and fluoroscopy, and were withdrawn. One catheter tip reported to be in the RA by ultrasound was found to be at the cavo-atrial junction according to fluoroscopy and was not repositioned (Table 4). Overall, inter-rater agreement between the modalities in our study is 0.925 with a p-value <0.001.

**Table 3:** Inference of chest X-ray with ultrasound RA: right atrium

Chest X-ray inference	Ultrasound inference			Total
	Above RA	Within RA	Below RA	
Above RA	5	0	0	5
Within RA	1	21	0	22
Below RA	0	0	3	3
Total	6	21	3	30

**Table 4:** Degree of agreement between ultrasonography and fluoroscopy at different levels

Site	Degree of agreement of ultrasonography with fluoroscopy (%)
Above right atrium	83.3
Right atrium	95.5
Right ventricle	100

In this study, the total number of radiological exposures required for confirming the position of VAD was 113 at an average of 3.76 exposures per patient. While eight children underwent continuous fluoroscopy (lasting three seconds or more) for catheter tip localization, 15 children were exposed to more than two still fluoroscopic shots to confirm the position of the VAD. The common reasons for multiple exposures were a lack of expertise of the radiographer, poor visibility of the tip, and poor communication between the radiographer and operating surgeon.

## Discussion

In the present era of point-of-care ultrasound, whose convenience and utility are well established, newer applications are being added to our armamentarium every day. Though there have been studies where sonography was attempted for locating the tip of short-term catheters in the ICU or ward setup [10], none of them have looked into the usage of ultrasound in locating the tip of long-term tunneled central venous catheters. The cavo-atrial junction is considered the accurate position for the tip to be in case of central lines. But long-term catheters like Hickman and Port-a-Cath, through which chemotherapeutic agents are to be infused, the proximal RA, in addition to the cavo-atrial junction, have been deemed acceptable positions [14].

In our institution, fluoroscopy is considered the reference standard for determining the final position of the catheter tip. Fluoroscopy has certain drawbacks. First, it exposes the child and the theatre personnel to ionizing radiation [3]. Second, it often leads to wastage of theatre time as organizing for fluoroscopy is a cumbersome process. A considerable amount of time is spent on waiting for the technician to come and on preparing the image intensifier for use [7]. In a high-volume centre such as ours, where operation lists are fully booked, long delays can lead to stress on the theatre staff to run long lists or cancellation of the last case on the list. Third, there is a lack of clarity on the best position to be used for fluoroscopy [13], and its ability to differentiate between the cavo-atrial and atrial location of the catheter tip.

In our study, we have attempted to extend the use of ultrasonography from just being a guide in the placement of a VAD by locating the vein and directing the needle to being the mainstay in confirming the tip of a placed long-term VAD. The position of the catheter tip has been proven to be the main independent prognostic factor for malfunction and reduced duration of usage of the device, and therefore, confirming the location of the catheter tip is of utmost importance in avoiding complications, especially in the long term, in sick children [11]. Since the placement of the catheter was performed mostly by an open approach, the chance of an intra-arterial positioning was negated, with confirmation required only for locating the catheter tip.

For determining the correct position of the catheter tip, we relied on quick administration of 2 mL saline through a 2-cc syringe, unlike Blans et al. [10], who administered agitated saline (saline mixed with air). We studied three parameters, namely time to visualization of opacification, shape, and site of opacification. While previous studies [10] utilized only the time to visualization of the jet and the site of the jet in determining the catheter tip, the addition of the shape of the jet in our study was useful, especially in two patients, where abnormality in the shape helped differentiate the site of the catheter tip.

However, expertise is required to perform the scan and interpret the findings [9,14]. Acquiring an adequate image without being able to move the patient on the table can be challenging. Sometimes other factors can hamper the acquisition of a good image, such as massive hepatomegaly, obesity, or a thoracic mass pushing the mediastinum. We encountered massive hepatomegaly and obesity hampering a good subxiphoid view, which entailed the usage of the alternative apical approach in two of our patients to view all four chambers. The remaining 28 patients had undergone subxiphoid view for optimal four-chamber views. In one of our patients, the jet was seen going into the RV and left atrium, indicating the presence of an atrial septal defect (ASD).

In our study, there was only one patient in which a discordance was found between fluoroscopic and sonographic assessment of the location of the catheter tip. While ultrasound assessed the catheter tip to be in RA, fluoroscopy determined it to be at the cavo-atrial junction but not requiring clinical manipulation. We observed the inter-rater agreement between the two modalities to be 0.925, which is encouraging, unlike Kamalipour et al., who noticed inter-rater agreement of 0.72 and concluded that contrast-enhanced ultrasonography cannot replace fluoroscopy [15].

Our study had a few limitations. First, as our sample size was small, and a proper validation of the results may require larger multi-institutional studies. Second, in our study, the ultrasonography was performed by experienced anaesthetists, and as sonography is highly operator dependent, it is imperative to impart proper training to the anaesthetists prior to independently performing diagnostic scanning [9,14]. Third, our reference standard was fluoroscopy, which is time-tested and easily available, but not very sensitive in identifying the correct position of the tip (the act of breathing can alter tip position by 1 cm, or in locating the tip within small veins like the azygos).

## Conclusions

There exists strong evidence to state that sonography is an accurate and safer diagnostic modality for confirming catheter tip position after the insertion of a long-term VAD. In this study, it was shown statistically that the catheter tip of the VAD can be correctly determined by ultrasonography, negating the need for fluoroscopy, which has its limitations, such as radiation exposure to both the patient and treating doctors, limited availability, higher costs, and logistical issues. Further large-scale studies are needed before considering it a gold standard for confirmation, and more age groups should be explored.
